# Improving Operating Room Efficiency: Relocating a Surgical Oncology Program Within a Health Care System

**DOI:** 10.31486/toj.22.0030

**Published:** 2022

**Authors:** James W. Orr, Ann M. Thompson, Dennis Bruens, Jennifer Higgins, Yvonne Rittenhouse, Mark Bloomston, Adam I. Riker

**Affiliations:** ^1^ Lee Health Regional Cancer Center, Fort Myers, FL; ^2^ GenesisCare USA, Fort Myers, FL

**Keywords:** *Efficiency*, *operating room*, *professional burnout*, *quality improvement*, *surgical oncology*

## Abstract

**Background:** To meet increased community and regional needs for quality services, our hospital system concluded that its established surgical oncology program—consisting of gynecologic oncology (4 physicians), surgical oncology (2 physicians), and otolaryngologic oncology (2 physicians)—would be best served by the transition of the comprehensive surgical oncology program to a new oncology-naive hospital. We describe the overall strategy and approach involved with this move, its implementation, operating room efficiency results, and physician satisfaction associated with the relocation.

**Methods:** The purpose of the systematic plan for relocation, which was developed and refined during the 2 years preceding the move, was to facilitate a collective awareness and understanding of important patient-centered concepts and essential workflow. All parties involved in direct patient cancer care participated in multiple workgroups to successfully transition the surgical oncology practice. Following the transition to the oncology-naive hospital, components of the operative cases and surgical data were prospectively collected for the initial 6 weeks and compared to retrospective data from the last 8 weeks at the established hospital. The surgical day for each surgeon was deconstructed, and measured variables included total surgical cases, total surgical hours, surgical minutes per case, total anesthesia hours, first case on-time surgical starts, surgical stretcher wheels out to surgical stretcher wheels in, surgical stretcher wheels out to next case start, case end to postanesthesia care unit (PACU), and case end to case start.

**Results:** Five hundred twenty-nine surgical cases encompassing 1,076 anesthesia hours and 710 surgical hours were completed during the 14-week evaluation period. The gynecologic oncologists completed the majority of surgical procedures in both settings. The percentage of first case on-time surgical starts initially decreased during the 6-week interval at the oncology-naive hospital, but interval subset analysis suggested a return to the pre-move norm. Surgical stretcher wheels out to surgical stretcher wheels in had a wide range (9 minutes to 305 minutes) for all surgical sections, but no statistically significant difference was seen overall or for any surgical section. Case end to PACU significantly increased for gynecologic oncology but not for surgical oncology or otolaryngologic oncology. Overall case end to case start times decreased nonsignificantly (63.7 ± 3.1 mean minutes vs 60.3 ± 1.7 mean minutes) following the move. A physician survey found that physicians’ expectations were met in terms of the move occurring smoothly without major issues, surgical scheduling and accommodation, anesthesia services, and surgical personnel. Physicians indicated less satisfaction with quality and availability of instrumentation.

**Conclusion:** The transfer of established surgical oncology services to an oncology-naive hospital was associated with early surgeon and operating room staff support, as well as process and programmatic alignment among stakeholders. The success of this transition required transparency, open and honest communication, and problem solving at all levels. The move of a surgical oncology program to an oncology-naive hospital was deemed successful without deterioration of time-related variables associated with operating room efficiency and physician satisfaction. The breakdown and analysis of key components of the surgical day offered additional opportunities for quality improvement in operating room efficiency.

## INTRODUCTION

The management of acute care surgical diagnoses and procedures constitutes a substantial health care cost nationally, representing nearly 25% of US government inpatient care expenditures.^1^ Efficient and safe operating room utilization is an important and necessary component of maintaining and improving upon the quality of surgical care, cost savings, and hospital net revenues.^2^

A chronic level of highly stressful clinical decision-making both in and out of the operating room epitomizes the intense, high-risk practice of a surgical oncologist. These surgeons not only manage a large volume of patients (many with multiple and significant comorbidities) but also face long work hours, on-call responsibilities, and the physical and mental demands of a busy operating schedule involving radical procedures that routinely necessitate life-or-death decision-making.^3^

According to the literature, daily workload and increased control over the work schedule are independent predictors that improve emotional resilience, provide a sense of personal accomplishment, and lessen the risk of physician and staff burnout.^3^ Consequently, any potential change in the physician work environment and work schedule should be approached carefully and undertaken thoughtfully to maintain continuity and best care practices, while minimizing workplace disruptions that may negatively impact physician performance.

A successful subspecialty surgical oncology platform involves the balanced coordination of highly skilled physicians (surgeons, anesthesiologists, pathologists, radiologists) and ancillary supportive staff (nurses, technicians, and administrative staff), schedule management, the essential physical environment, and easy access to necessary instrumentation. Administrative or project development support, an excellent communication system, and access to data and evidence review are critical components for clinical success.^4^

For a health care organization to plan and execute the complete physical relocation of a mature surgical oncology program from one hospital to another is a quite uncommon occurrence, especially when the relocation hospital is not set up to deliver oncology services in the operating theater. No published literature examines the aspects of planning and quality outcomes associated with such a transfer of surgical services. Obviously, limiting the risk of poor surgical outcomes (eg, complications) and maintaining physician, staff, and patient satisfaction should be heavily weighted, as all stakeholders and key leadership must be fully engaged to optimize the opportunity for success in this unusual clinical and management scenario.

We describe the strategy for and report the results of relocating an established, mature surgical oncology program to an oncology-naive hospital setting.

## PLANNING AND IMPLEMENTATION

Lee Health's inpatient surgical oncology program, including gynecologic oncology (4 physicians), surgical oncology (2 physicians), and otolaryngologic oncology (2 physicians), was established and expanded during a 22-year period at Lee Memorial Hospital in Fort Myers, Florida. Surgeons had been recruited to this program during this period, with the last surgeon joining just prior to the physical move. None of the surgeons was hospital-employed; all were independently employed by GenesisCare USA. Because Lee Memorial Hospital was an aging facility that restricted future growth, Lee Health developed a strategic plan to relocate the surgical oncology service to Gulf Coast Medical Center, a sister hospital that had been recently expanded and was located 7.3 miles from Lee Memorial Hospital. At the time of the move, Gulf Coast Medical Center was considered naive to the needs of a surgical oncology program. The systematic plan for relocation, developed and refined during a 2-year period (2018-2019), was created to facilitate collective awareness and understanding of the important concepts and essential work steps.

All parties involved in cancer care were invited to establish and join the culture deemed necessary to successfully transition the surgical oncology practice to the new hospital setting. The unambiguous goal was to identify and re-create accepted, successful, evidence-based care pathways and workflow efficiencies and to identify, modify, and improve upon those considered suboptimal. The relocation was initially planned for early May 2020 but occurred in August 2020 during the coronavirus disease 2019 (COVID-19) pandemic.

Preparation for the move included the following:

1. Beginning in spring 2018, 1-day, offsite cancer center retreats were held at approximately 8-month intervals (3 retreats) to develop a collective vision and set of goals and expectations for the cancer program, including all aspects of acute care services.
  - All oncology-specific physicians (including surgery, pathology, radiology, and anesthesia) were invited, and most attended. Active participation was encouraged. Open dialog invited all comments; none were censured.
  - Representative nursing and operating room staff, as well as support personnel (pharmacy, rehabilitation, genetics, social service), attended and actively participated.
  - Senior administrative leadership from all levels attended and candidly demonstrated their commitment. Open dialog was the rule.
  - Collectively, this group developed, refined, and continually modified a comprehensive list of “must have,” “priority have,” and “nice-to-have” opportunities and preemptively created and managed expectations.
  - Thorough minutes of each meeting were disseminated to all stakeholders shortly after each meeting. Comments, additions, and clarifications were encouraged and incorporated.
2. Within weeks after the initial retreat, systematic, separate, multiple meetings with surgeons (collectively, within sections, and individually), operating room nurses and staff, anesthesia staff, and pathology staff were convened to discuss the transfer of block time and operating room time to mitigate concerns.
  - Surgeons were guaranteed to keep their current operating room start times, schedules, and block time at Gulf Coast Medical Center.
  - Existing oncologic pathology expertise was also guaranteed to relocate with appropriate facilities and support.
  - All Lee Memorial Hospital surgical oncology team members (including operating room technicians and nurses) were offered a transfer opportunity to Gulf Coast Medical Center. Approximately 60% accepted. No increased compensation was involved with the decision to participate or not.
  - A master list of necessary instrumentation and equipment was developed to either purchase or move from Lee Memorial Hospital to Gulf Coast Medical Center. All surgical preference cards were reviewed and updated.
3. Regular working meetings with the Gulf Coast Medical Center staff, and quarterly, monthly, and later, weekly calls occurred to address general or specific issues or concerns regarding anesthesia, operating rooms, pathology, hospitalists, intensivists, radiologists, and the emergency department to create an oncology-specific culture and workflow, addressing the most minute details (eg, the use of shoulder restraints for minimally invasive procedures). The electronic medical record (EMR) did not change, as Epic (Epic Systems Corporation) was the established EMR at both facilities.
4. To engage personnel at all levels in the vision, personal tours of the Gulf Coast Medical Center facility were scheduled during construction and remodeling. Constructive feedback was incorporated into the design plans whenever possible.

## EVALUATION PERIODS AND OUTCOME VARIABLES

Following the move, the components of the surgical day were determined, and the surgical data were prospectively collected for the initial 6 weeks (August 17, 2020, to September 29, 2020) at Gulf Coast Medical Center and compared to the retrospective data from the last 8 weeks (June 19, 2020, to August 19, 2020) at Lee Memorial Hospital. Notably, no restrictions were placed on performing elective or oncologic procedures during the interval of evaluation despite the COVID-19 pandemic. Robotic surgical data were excluded from the analysis as this technology was relatively new and evolving in the surgical oncology and otolaryngologic oncology programs. Measured variables included total surgical cases, total surgical hours, surgical minutes per case, total anesthesia hours, first case on-time surgical starts, surgical stretcher wheels out to surgical stretcher wheels in, surgical stretcher wheels out to next case start, case end to postanesthesia care unit (PACU), and case end to case start. The mean, median, and range times for each of these variables were calculated and tabulated by individual surgeon and aggregated to specific surgical sections. Statistical analyses were undertaken with one-way analysis of variance (alpha a priori <0.05, Excel 2016 [Microsoft Corporation]).

Post-move data were analyzed collectively at 4-week and 6-week intervals to detect any positive or negative changes on operative efficiency that might have occurred early or late in the process. The 6-week data were used for this report, and any clinical or statistical changes between the 4- and 6-week analyses are reported.

Physicians were asked to complete a satisfaction survey regarding meeting relocation expectations 8 weeks following the move.

## RESULTS

We evaluated 529 surgical cases encompassing 1,076 anesthesia hours and 710 surgical hours completed during the 14-week evaluation period (Table 1). In the overall analysis of the combined data from all surgical sections, we found no clinical or statistical difference in either the mean (37.9 vs 37.7, respectively) or median (36.5 vs 36.0, respectively) number of cases completed weekly at Lee Memorial Hospital and Gulf Coast Medical Center or in surgical or anesthesia hours.

**Table 1.** Operative Data by Hospital^a^

Variable	Lee Memorial Hospital, June 19, 2020-August 19, 2020	Gulf Coast Medical Center, August 17, 2020-September 29, 2020
Total cases	303	226
Cases per week, median (range)	36.5 (32-46)	36.0 (32-45)
Cases per week, mean ± standard error	37.9 ± 1.8	37.7 ± 1.9
Total anesthesia hours	577	499
Anesthesia hours per case	1.9	2.2
Total surgical hours	397	313
Surgical hours per case	1.3	1.4

^a^Analysis of variance difference for all variables was not significant, *P*=0.173074.

### Surgical Minutes per Case

Evaluation of the 3 surgical specialties combined showed no significant difference in surgical minutes per case, with a total of 78.6 mean surgical minutes per case at Lee Memorial Hospital and 81.7 minutes at Gulf Coast Medical Center (Table 2). Analysis by surgical section revealed no postrelocation difference in surgical minutes per case for gynecologic oncology; however, surgical minutes per case significantly increased following the move for the other 2 sections. For surgical oncology, mean surgical minutes per case were 83.9 minutes at Lee Memorial Hospital vs 104.9 minutes at Gulf Coast Medical Center. For otolaryngologic oncology, mean surgical minutes per case were 167.0 minutes at Lee Memorial Hospital vs 230.0 minutes at Gulf Coast Medical Center (*P*<0.05).

**Table 2.** Mean and Median Surgical Minutes per Case by Section and Overall

	Surgical Minutes per Case, Mean ± SE		Surgical Minutes per Case, Median (Range)	
Section	Lee Memorial Hospital	Gulf Coast Medical Center	Lee Memorial Hospital	Gulf Coast Medical Center
Gynecologic oncology	58.6 ± 2.6	57.6 ± 3.0	53 (6-197)	51 (2-279)
Surgical oncology	83.9 ± 9.7	104.9 ± 11.7^a^	57 (5-315)	88.5 (11-314)
Otolaryngologic oncology	167.0 ± 25.3	230.0 ± 46.2^a^	114 (9-727)	174 (28-622)
Overall	78.6 ± 4.8	81.7 ± 5.9	57 (5-727)	58 (2-622)

^a^One-way analysis of variance difference between the mean at Lee Memorial Hospital and the mean at Gulf Coast Medical Center is significant, *P*<0.05.

Gynecologic oncology performed the most surgical procedures in both settings (65% at Lee Memorial Hospital and 71% at Gulf Coast Medical Center) and accounted for the most total surgical minutes (43% at Lee Memorial Hospital and 48% at Gulf Coast Medical Center). The median number of gynecologic oncology cases per week increased by 12.6% (25.3 vs 28.5 cases per week) following the relocation. No clinically significant difference (<5%) was seen in the number of gynecologic oncology minor procedures (cervical conization, molar evacuation, partial vulvectomy, hysteroscopy, dilatation and curettage) performed after the move. The number of surgical oncology procedures did not change significantly; however, the mean surgical minutes per case increased by 25% (*P*<0.05) following the move. Although the number of otolaryngologic oncology procedures decreased by 43% (47 vs 27 cases) during the first 6 weeks at Lee Memorial Hospital, mean surgical minutes per case increased by 38% (*P*<0.05).

### First Case On-Time Surgical Starts

Overall, a clinical but not statistically (*P*>0.064) significant decrease occurred in the percentage of first case on-time surgical starts following the move (Table 3). However, analysis of data from the 5th and 6th week after the move showed a marked improvement in the proportion of first case on-time surgical starts for the largest service (gynecologic oncology): 92% at weeks 5 and 6 vs 76% overall for weeks 1 through 6. No 5th and 6th week differences were found in any of the other time variables analyzed.

**Table 3.** Percentage of First Case On-Time Surgical Starts by Section and Overall

	First Case On-Time Surgical Starts, %	
Section	Lee Memorial Hospital	Gulf Coast Medical Center
Gynecologic oncology	100	76^a^
Surgical oncology	76	57
Otolaryngologic oncology	65	79
Overall	89	73^b^

^a^92% for weeks 5 and 6.

^b^*P*>0.064.

### Surgical Stretcher Wheels Out to Surgical Stretcher Wheels In

Analysis of surgical stretcher wheels out to surgical stretcher wheels in indicated a wide range (9 minutes to 305 minutes) for all surgical sections in both hospitals. However, we found no significant difference in surgical stretcher wheels out to surgical stretcher wheels in mean times between sections or between hospitals (Table 4). The greatest difference was seen in a trend toward improvement with surgical oncology: the mean surgical stretcher wheels out to surgical stretcher wheels in time at Lee Memorial Hospital was 62.1 minutes vs 42.4 minutes at Gulf Coast Medical Center. While this decrease suggests clinical improvement, the difference did not reach statistical significance.

**Table 4.** Surgical Stretcher Wheels Out to Surgical Stretcher Wheels In by Section and Overall

	Surgical Stretcher Wheels Out to Surgical Stretcher Wheels In, Minutes, Mean ± SE		
Section	Lee Memorial Hospital	Gulf Coast Medical Center	*P* Value
Gynecologic oncology	28.6 ± 2.5	27.3 ± 1.3	0.658484
Surgical oncology	62.1 ± 15.1	42.4 ± 7.0	0.411795
Otolaryngologic oncology	24.2 ± 2.0	25.5 ± 3.6	0.770894
Overall	33.8 ± 3.3	28.8 ± 1.4	0.22928

### Surgical Stretcher Wheels Out to Next Case Start

A wide range (11 minutes to 319 minutes) of times was seen for surgical stretcher wheels out to next case start. Surgical stretcher wheels out to next case start decreased for surgical oncology (means of 83.7 minutes vs 62.4 minutes) and increased for otolaryngologic oncology (means of 49.3 minutes vs 56.0 minutes) following the move to Gulf Coast Medical Center, but these differences were not statistically significant (Table 5). For all surgical sections combined, the means for surgical stretcher wheels out to next case start following the move to Gulf Coast Medical Center were not significantly different (54.3 minutes vs 50.1 minutes).

**Table 5.** Surgical Stretcher Wheels Out to Next Case Start by Section and Overall

	Surgical Stretcher Wheels Out to Next Case Start, Minutes, Mean ± SE		
Section	Lee Memorial Hospital	Gulf Coast Medical Center	*P* Value
Gynecologic oncology	48.2 ± 2.4	47.9 ± 1.4	0.922881
Surgical oncology	83.7 ± 15.3	62.4 ± 7.6	0.341063
Otolaryngologic oncology	49.3 ± 2.6	56.0 ± 4.3	0.231824
Overall	54.3 ± 3.2	50.1 ± 1.5	0.34101

### Case End to Postanesthesia Care Unit

We also evaluated the time from case end (skin closure) to PACU. For gynecologic oncology, this time significantly increased from a mean of 9.2 minutes at Lee Memorial Hospital to a mean of 11.7 minutes at Gulf Coast Medical Center (Table 6). However, case end to PACU time did not significantly change for surgical oncology or otolaryngologic oncology. Overall, the mean case end to PACU time increased significantly from 10.2 minutes to 12.5 minutes after the move to Gulf Coast Medical Center.

**Table 6.** Case End to Postanesthesia Care Unit by Section and Overall

	Case End to Postanesthesia Care Unit, Minutes, Mean ± SE		
Section	Lee Memorial Hospital	Gulf Coast Medical Center	*P* Value
Gynecologic oncology	9.2 ± 0.3	11.7 ± 0.8	0.001206
Surgical oncology	12.0 ± 0.7	13.7 ± 1.2	0.202791
Otolaryngologic oncology	12.5 ± 1.2	16.2 ± 1.6	0.079044
Overall	10.2 ± 0.3	12.5 ± 0.6	0.000722

### Case End to Case Start

Assessment of case end (skin closure) to case start (incision) times revealed a large range (15 minutes to 327 minutes). However, analysis of the overall mean times showed a slight nonsignificant decrease (63.7 minutes vs 60.3 minutes) after the move to Gulf Coast Medical Center (Table 7).

**Table 7.** Case End to Case Start by Section and Overall

	Case End to Case Start, Minutes, Mean ± SE		
Section	Lee Memorial Hospital	Gulf Coast Medical Center	*P* Value
Gynecologic oncology	48.2 ± 2.4	47.9 ± 1.4	0.922881
Surgical oncology	83.7 ± 15.3	62.4 ± 7.6	0.341063
Otolaryngologic oncology	49.3 ± 2.6	56.0 ± 4.3	0.231824
Overall	63.7 ± 3.1	60.3 ± 1.7	0.304101

### Physician Satisfaction Survey

The physician survey administered after the move suggested that physicians’ expectations were met at the new location (Table 8). Overall, physicians responded that the move to Gulf Coast Medical Center occurred smoothly without any major issues identified (mean score of 4.57 on a 5-point scale). Surgical scheduling and accommodation (4.57); anesthesia services, including start times, turnover, attitude, and availability (4.85); and surgical personnel, including operating room nurses and surgical technicians (4.71) were rated highly. Physicians expressed less satisfaction (3.42) with the quality and availability of instrumentation.

**Table 8.** Physician Satisfaction Survey Results by Section and Overall

Question	Gynecologic Oncology	Surgical Oncology	Otolaryngologic Oncology	Overall
Surgical scheduling/accommodation met expectation	5.0	3.5	5.0	4.57
Anesthesia services (start times/turnover/attitude/availability) met expectation	4.75	4.5	5.0	4.85
Instrumentation (quality/availability) met expectation	3.75	2.5	4.0	3.42
Surgical personnel (operating room nurses/surgical technicians) met expectation	4.75	5.0	4.0	4.71
Overall, the move to Gulf Coast Medical Center occurred smoothly without major issues	4.75	4.0	5.0	4.57

Notes: Possible responses were on a 5-point Likert scale: 1, strongly disagree; 2, disagree; 3, agree; 4, agree for the most part; 5, strongly agree. Data are reported as mean scores.

## DISCUSSION

In an established surgical oncology program, oncologic surgical procedures are used throughout the cancer care journey: for diagnosis and staging, curative therapy, posttreatment surveillance, management of recurrence, and palliative indications. Delivering optimal oncologic surgical outcomes is one of the highest priority goals for a comprehensive surgical oncology program. The best formula for success is to engage technically capable surgeons and operating room staff and to offer efficient and suitable surgical times, appropriate instrumentation, and adequate space to maintain and grow surgical programs.

Few hospital systems have the desire or strategic need to relocate a major, mature surgical oncology program, and the prospect can be daunting for hospital leadership. However, because of the rapidly growing need in our community for high-quality cancer care, additional hospital beds had to be dedicated for patients with cancer. Consequently, because the existing program was located in an aging hospital with physical growth limitations, we embarked upon such a relocation. We realized the magnitude of such a move and the challenges it would create. Our goal was to minimize the interruption or disruption of oncologic surgical services in this high-stress environment to help avoid physician and staff burnout and its effect on physician productivity, staff satisfaction, and patient care.

Early in the planning process, numerous stakeholders expressed concerns regarding a move-related increase in inefficiency and physician and staff dissatisfaction. There was also much administrative angst over the increased costs associated with additional operating room staffing needs. Additional cost was recognized as a legitimate and common concern in surgery suites that can result in additional stress to physicians and staff, dissatisfaction for patients, and financial loss to hospitals.^5^ The overall mindset surrounding this move was based in transparency, understanding that priority would be placed on managing the work time and the number of patients while optimizing throughput with the intent to increase patient, staff, and physician satisfaction.

The day-long retreats during the planning phase were devoted to the development of the vision and goals for the cancer program. Although attendance was not mandatory, the entire clinical staff was encouraged to be a part of the solution. We knew that if all stakeholders were not aligned, we had little reason to believe that the relocation could be implemented successfully. The final retreat was devoted to the location and timing of the move, as well as collectively developing consensus around the programmatic resources and elements.

The strongest predictor of work-life balance and burnout is physicians’ control over their schedules and hours worked.^3,6^ A key event occurred early in the planning phase when the oncologic surgeons were guaranteed that they would retain their identical start and block operating room times and that the existing surgical pathology expertise would be transferred concurrently. Those involved in the move unanimously agree that because of these guarantees, the independently employed surgical oncologists believed that they could trust the process and began to identify themselves as the cultural architects who could safely become champions of the relocation.

Effective, efficient communication throughout the continuum of health care is critically important. The goal of planned and sometimes spontaneous communications was to systematically define, institute, and refine clinical care pathways and workflow efficiencies. The ongoing monthly, and later weekly, meetings and calls involved subspecialty group and one-on-one discussions, fertilizing cross-cultural and cross-specialty communication with timely feedback. The late part of this process occurred during the COVID-19 pandemic, and the use of electronic communications and virtual meetings likely increased participation. Virtual meetings were used extensively to facilitate inclusion and minimize time demands for stakeholders.

Operating room efficiency is directly dependent upon throughput. Operating room staff, nurses, and anesthesiologists typically receive the same compensation and leave work at the same time, regardless of the number of procedures that are performed or their outcomes. On the other hand, surgeons are typically compensated on volume or the quality outcomes of the operations performed. Thus, the magnitude (529 surgical cases, 1,076 anesthesia hours, and 710 surgical hours) of this move invited a review to determine the effectiveness of the preparatory process and to identify move-related deficiencies for a service that completes >2,000 cases involving >4,000 anesthesia hours annually.

Typically, operating room efficiency and success are measured by sustaining a high percentage of first case on-time surgical starts and short turnover times. Both variables are the subject of numerous research articles and operating room committee discussions. For our analysis, we further deconstructed the components of the surgical day to recognize and evaluate additional areas of potential inefficiency and opportunities for improvement (Figure).

**Figure.**
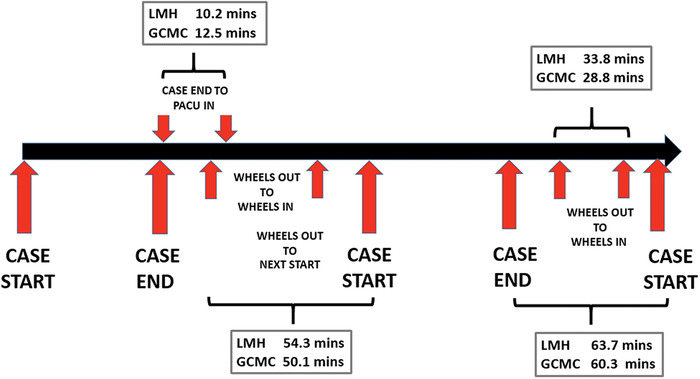
**Anatomy of the surgical day showing overall mean times at Lee Memorial Hospital (LMH) and Gulf Coast Medical Center (GCMC).** mins, minutes; PACU, postanesthesia care unit.

Critics might question the validity of this data as the latter part of preparation and the physical move occurred during the COVID-19 pandemic. The absence of any overall differences in cases per week, total anesthesia hours, anesthesia hours per case, and surgical hours per case are likely a true reflection of the actual routine of our surgical oncology program. In the state of Florida, local and state COVID-19–related decisions did not disrupt the delivery of surgical oncology care. Both hospitals permitted all our oncology surgeons’ cases to be completed as scheduled without delay during the pre- and post-move intervals reported.

Our comparative overall and surgical section analyses of operating room efficiency variables suggest the opportunity to uncover meaningful clinically or statistically significant surgical section differences that may not be detected if only an overall measurement is examined. Assessment by surgical sections (and their different needs and routines) may uncover opportunities and provide direction for improvement. In this report, the mean surgical minutes per case (Lee Memorial Hospital 78.6 ± 4.8 minutes vs Gulf Coast Medical Center 81.7 ± 5.9 minutes) for the 3 services did not differ; however, the individual section analysis revealed a statistically and clinical significant prolongation of 25% in surgical oncology cases (Lee Memorial Hospital 83.9 ± 9.7 mean minutes vs Gulf Coast Medical Center 104.9 ± 11.7 mean minutes) and 38% in otolaryngologic oncology cases (Lee Memorial Hospital 167.0 ± 25.3 mean minutes vs Gulf Coast Medical Center 230.0 ± 46.2 mean minutes). These differences may be related to a number of factors, including a short delay in scheduling ultraradical procedures during the last days at Lee Memorial Hospital or other factors associated with case mix, such as insurance issues or patient preference.

No information suggests a different case mix in surgical oncology and otolaryngologic oncology after the relocation. Importantly, a review of gynecologic oncology cases indicated that no difference in the proportion of minor cases occurred after the move, suggesting a successful programmatic transfer. Other potential factors leading to longer operating room times include new instrumentation needs and unfamiliarity of the intraoperative staff with procedures at the new hospital. To this point, the lowest satisfaction score on the survey was from surgical oncology for instrumentation issues, suggesting a possible cause of case prolongation following the move. Regardless, this information invites an in-depth analysis to determine the etiology of these increased times and continued monitoring for improvement.

The on-time surgical start of the first case of the day is an important operating room efficiency and quality metric. First case surgical start delays can adversely affect surgeons, staff, and patient satisfaction and create further delays throughout the surgical day. First case on-time surgical starts in oncology procedures are most likely to occur when solo anesthesia staffing is present, as additional anesthesia supervisory duties detract from time attentiveness.^7^ However, anesthesia availability, surgeon availability, and patient availability or need for additional patient evaluation (eg, blood work, consent) have been reported to contribute equally to favorable and adverse effects for this metric.^8^ Interestingly, at least 1 report suggests that patient systemic disease as defined by the American Society of Anesthesiologists Physical Status Classification has little effect upon first case on-time surgical starts.^9^

Our data suggest a clinically but not statistically significant decreased percentage of on-time surgical starts following the relocation. In the 8 weeks preceding the move, the percentage of overall first case on-time surgical starts was 89%, with gynecologic oncology at 100%. In the 6 weeks after the move, the percentage of overall first case on-time surgical starts decreased to 73%. While the exact factors related to this decrease are unknown, we were encouraged to note that contemporaneous communication between gynecologic oncology and anesthesia was followed by 92% first case on-time surgical starts in weeks 5 and 6 postrelocation. This communication line was likely assisted by the extent of pre-move physician-to-physician communication. Additionally, one gynecologic oncology surgeon maintained his own records confirming this trend (personal communication). Regardless, our information suggests that any future analyses of first case on-time surgical starts should include both an overall and a surgical section evaluation to reveal opportunities for improvement.

Turnover time, composed of multiple elements, remains the classic variable measured to quantify day-to-day operating room efficiency. Measurements of the components—surgical stretcher wheels out to surgical stretcher wheels in, surgical stretcher wheels in to next case start, and case end to PACU—offer a complete dissection and facilitate understanding. However, surgeons might consider the actual time from case end to case start as a better measure as it correlates with actual surgeon downtime. While surgeons often fill this time with other activities (eg, hospital rounds, answering calls, preparation for the next case), establishing a culture that respects all stakeholders’ time can only increase physician, staff, and patient satisfaction.

Most professional organizations, including the Association of periOperative Registered Nurses, use surgical stretcher wheels out to surgical stretcher wheels in, which includes case end to PACU and next case preparation, as a measurable metric representing turnover time. Our evaluation of overall surgical stretcher wheels out to surgical stretcher wheels in mean times suggested an important clinical (5 minutes) but not statistical decrease following the relocation: 33.8 ± 3.3 minutes at Lee Memorial Hospital vs 28.8 ± 1.4 minutes at Gulf Coast Medical Center. These overall times were at the lower end or better than the times reported by Dexter et al from 4 tertiary care hospitals (34-66 minutes).^10^ Many factors contribute to prolongation of turnover time, with as much as 44% of a facility's total available operative time being nonoperative.^11^ Attending surgeon presence in the operating room within 10 minutes of a patient's arrival was found to significantly decrease time to incision by 33%.^12^ Therefore, culture, staff-physician alignment, and establishing expectations are of paramount importance in a transfer of services, and the introduction of new factors (eg, a new EMR)^13^ and inefficient processes that may contribute to prolongation should be minimized or avoided.

The mean times for case end to surgical stretcher wheels out (case end to case start [Table 7] minus surgical stretcher wheels out to next case start [Table 5]) were not significantly different between hospitals: 9.4 minutes at Lee Memorial Hospital vs 10.2 minutes at Gulf Coast Medical Center). However, further analysis of the turnover time after the move revealed a small clinically significant increase in the case end to PACU variable (Table 6). In our current workflow, this process requires the presence of the operating room circulating nurse, and the short time increase may be related to the new environment and new personnel. However, this time increase did not adversely affect the overall surgical stretcher wheels out to next case start times. In fact, review of the surgical stretcher wheels out to next case start time showed a nonstatistically significant decrease after the move: 54.3 ± 3.2 mean minutes at Lee Memorial Hospital vs 50.1 ± 1.5 mean minutes at Gulf Coast Medical Center. However, the ranges for this variable were very wide at Lee Memorial Hospital (11-319 minutes) and Gulf Coast Medical Center (13-152 minutes).

Oncologic surgeons desire to be efficient and productive as they balance their daily workload. They might consider case end to case start to be a valid measure of their downtime in the operating room, and a physical move that potentially increases this downtime is not likely to be accepted. Our analysis found a nonstatistically significant decrease in this variable after the move (63.7 ± 3.1 mean minutes at Lee Memorial Hospital vs 60.3 ± 1.7 mean minutes at Gulf Coast Medical Center), again suggesting the benefit of the preparatory process.

A true measure of the success of this process is undeniably related to physician satisfaction. Overall, our results indicated that expectations set by the surgeons a priori for a smooth transition were met. Interestingly, as previously noted, the lowest score across sections related to the availability and the quality of instrumentation. Surgical oncology reported the lowest satisfaction score on instrumentation issues. In our system, this service handles a wide variety of cases, resulting in variation in instrumentation needs. Arguably, this variation may have contributed to the significant increase in surgical oncology surgical case time, although these data are not specifically available. We recognize the importance and value of proper instrumentation and preparation, and this finding uncovered an opportunity for improvement in the day-to-day workflow in surgical oncology.

## CONCLUSION

The relocation of a surgical oncology program to an oncology-naive hospital was successful, and our analysis identified opportunities for quality improvement in the operating room milieu. Measurement of wide or long variables (ie, surgical stretcher wheels out to surgical stretcher wheels in) may obscure possibilities for true quality improvement. Our planned approach for relocation incorporated transparency, open and respectful communication, feedback on the process, and flexibility in plan implementation. As such, relocation resulted in enhanced operating room efficiencies and in improvement of several measured parameters.

## Notes

*This article meets the Accreditation Council for Graduate Medical Education and the American Board of Medical Specialties Maintenance of Certification competencies for Patient Care, Medical Knowledge, and Systems-Based Practice.*
